# Dose–Response Between Serum Prealbumin and All-Cause Mortality After Hepatectomy in Patients With Hepatocellular Carcinoma

**DOI:** 10.3389/fonc.2020.596691

**Published:** 2021-01-11

**Authors:** Rong-Rui Huo, Hao-Tian Liu, Zhu-Jian Deng, Xiu-Mei Liang, Wen-Feng Gong, Lu-Nan Qi, Xue-Mei You, Bang-De Xiang, Le-Qun Li, Liang Ma, Jian-Hong Zhong

**Affiliations:** Hepatobiliary Surgery Department, Guangxi Liver Cancer Diagnosis and Treatment Engineering and Technology Research Center, Guangxi Medical University Cancer Hospital, Nanning, China

**Keywords:** all-cause mortality, dose–response relationship, hepatocellular carcinoma, serum prealbumin, prognosis

## Abstract

**Background:**

The relationship between serum prealbumin and the risk of all-cause mortality after hepatectomy in patients with hepatocellular carcinoma (HCC) needs to be evaluated.

**Methods:**

We conducted a retrospective study. A Cox proportional hazards regression model was used to adjust for potential confounders. Prealbumin level was transformed by Z-scores and categorized into quartiles (Q1: <147 mg/L, Q2: 147–194 mg/L, Q3: 194–239 mg/L, Q4: >239 mg/L). We assessed the dose-response relationship between serum prealbumin and the risk of all-cause mortality using a restricted cubic spline model.

**Results:**

Data were included from 2,022 HCC patients who underwent hepatectomy at Guangxi Medical University Cancer Hospital in China between January 2006 and January 2016. The adjusted hazard ratios (HRs) for increasing quartiles of serum prealbumin were 0.78 [95% confidence interval (CI): 0.64–0.95] for Q2, 0.66 (0.53–0.81) for Q3, and 0.51 (0.41–0.64) for Q4 in the Cox model (all P < 0.001). Serum prealbumin showed an L-shaped, non-linear dose-response relationship with the risk of all-cause mortality (P < 0.001). Among patients whose serum prealbumin was below 250 mg/L, risk of all-cause mortality decreased by 27% (95% CI: 18–36%) per increase of one standard deviation (69.8 mg/L) in serum prealbumin.

**Conclusions:**

Levels of serum prealbumin under 250 mg/L may be considered dangerous with respect to all-cause mortality after hepatectomy in HCC patients. Serum prealbumin may be useful as a prognostic marker in HCC patients undergoing hepatectomy.

## Highlights

- Low serum prealbumin is associated with higher risk of all-cause mortality after hepatectomy in patients with hepatocellular carcinoma.
- Among these patients, levels of serum prealbumin under 250 mg/L may indicate elevated risk of all-cause mortality.

## Introduction

Hepatocellular carcinoma (HCC) is a common malignant neoplasm (1). Hepatectomy is one of the main radical treatments for HCC. The 5-year recurrence rate of HCC is up to 70% (2), and the 5-year overall survival (OS) is only 37% for patients with portal hypertension, 30% for those with multiple tumors, and 18% for those suffering macrovascular invasion after hepatectomy (3). Therefore, it is important to explore reliable preoperative risk markers to predict the OS of such patients. Some preoperative variables, such as tumor number, tumor differentiation, tumor size, alpha-fetoprotein (AFP) level, liver function, and the presence of liver cirrhosis have been associated with OS of HCC patients after hepatectomy (4–7). Preoperative malnutritional status may also contribute to poor prognosis after hepatectomy (8–10).

Serum prealbumin synthesized by the liver is a common laboratory indicator of nutritional status. Serum prealbumin is less affected by liver diseases than other serum proteins (11, 12). In addition, the level of serum prealbumin is not significantly altered by blood transfusion or supplemental infusion of human albumin. Some studies reported a positive association between the level of serum prealbumin and prognosis in various cancers (13–15). However, few studies have investigated the association between the level of serum prealbumin and the risk of mortality after hepatectomy in patients with HCC. In our previous study, we found that serum prealbumin <200 mg/L was associated with mortality after hepatic resection in patients with HCC (16). Other studies found similar results (17, 18). However, these studies were limited to multivariate analysis of serum prealbumin treated as a categorical variable, so they could not determine whether the risk of death of patients with HCC changed to different degrees with slight changes in serum prealbumin level.

In the present study, we examined the dose-response relationship of serum prealbumin level with the risk of all-cause mortality after resection in patients with HCC. In order to capture the relationship in detail and to compensate for the statistical problems of treating serum prealbumin as a continuous or categorial variable (19, 20), we applied a restricted cubic spline (RCS) function. The RCS function has proven effective at characterizing dose-response correlations between a continuous exposure (such as serum prealbumin) and all-cause mortality, regardless of whether the relationship was linear or not (21). It has been used in several single studies and meta-analyses (22–24). Therefore, in this study, we evaluated the dose-response relationships of serum prealbumin level with the risk of all-cause mortality after resection in patients with HCC.

## Methods

### Patients

We retrospectively reviewed records of patients who underwent hepatectomy for HCC between January 2006 and January 2016 at Guangxi Medical University Cancer Hospital, in Nanning, China. Eligible patients had to be admitted for initial HCC treatment at our hospital and had to have histologically confirmed HCC. Indications for hepatectomy was described as previous (2). No age restriction was applied during enrollment of patients in this study. HCC patients with distant metastasis or with other tumors were excluded, as were patients for whom preoperative serum prealbumin data were missing. Liver function indicators were assayed in blood collected at 6:00–7:00 a.m. on the second morning of hospitalization after overnight fasting. Hepatectomy was performed after 3 to 7 days of hospitalization. This study was conducted in accordance with the Declaration of Helsinki guidelines, and the protocol was approved by the Ethics Commitment of our hospital.

### Assessment of Covariates

The following data were extracted from patient records: sex, age, liver function categorized according to Child-Pugh category criteria, Barcelona Clinic Liver Cancer (BCLC) stage, serum prealbumin, hepatitis B surface antigen (HBsAg), albumin (ALB), aspartate aminotransferase (AST), alanine aminotransferase (ALT), total bilirubin (TBIL), AFP, and pathology of HCC involving cirrhosis, tumor size and number, integrity of tumor capsule, and presence or absence of macrovascular invasion. All measurements were obtained before surgery. However, liver cirrhosis and macrovascular invasion were confirmed by postoperative histopathology.

In order to ensure the accuracy and reliability of data, measures for standard quality controls were planned before the start of study, such as a table for collection of patients’ information and a code for replacement of categorical data. Two researchers independently collected and organized data; any discrepancies were corrected by a third reviewer.

### Outcomes and Follow-Up

Follow-up was conducted starting from the first month after surgery, every 2 months during the first year, then every 6 months thereafter until May 31, 2019 or death or loss to follow-up. During follow-up appointments, data were collected about time to HCC recurrence, survival or death, and liver function. Follow-up was conducted by telephone and review of the hospital’s data management system. The endpoint of the study was OS, defined as the interval from the date of hepatic resection until the date of last follow-up (May 31, 2019) or death.

### Statistical Analysis

Before testing independent associations of serum prealbumin with the risk of all-cause mortality in patients with HCC, Z-scores were calculated, such that the resulting estimated effect size indicated the change in serum prealbumin in terms of the standard deviation (SD). The Z-score normalizes the mean parameter value to 0 and the parameter value at one SD to 1. In addition, we also categorized serum prealbumin into quartiles based on sample size. Other categorical covariates were classified based on clinical findings and were entered as dummy variables. Continuous covariates were transformed into categorical variables based on cut-off values routinely used in clinical practice.

We assessed potential associations between serum prealbumin (as a categorical variable) and other characteristics using a chi-squared test or Fisher’s exact test. Survival curves were generated using the Kaplan-Meier method. The OS curves were compared between groups using the log-rank test. We tested associations of serum prealbumin with the risk of all-cause mortality using two Cox proportional hazards regression models: model 1 was adjusted for age and sex, while model 2 adjusted for age, sex, tumor size and number, tumor capsule, AFP, ALB, AST, ALT, TBIL, HBsAg, liver cirrhosis, liver function, and BCLC stage. From each fitted model, we searched for a linear trend by modeling the median value of each quartile to test ordered relations across the different serum prealbumin quartiles. Interactions between serum prealbumin and other factors were also examined using a likelihood ratio test, with a comparison of the log likelihood of the two models with or without the interaction terms. We calculated hazard ratios (HRs) for risk of all-cause mortality and the corresponding 95% confidence intervals (CIs).

We assessed the dose-response relationship between serum prealbumin (as a continuous variable) and the risk of all-cause mortality using RCS models. RCS assumes that the effect of the exposure on the outcome is a smooth, piecewise, cubic polynomial with linear tails (21). RCS models provide flexibility in fitting highly curved relationships, avoiding significant influences from outlying variables, and may provide better power than dichotomizing continuous variables (21). We used RCS models adjusted for the same potential confounders as in model 2, and plotted smooth curves with five knots at the 5^th^, 25^th^ (reference level), 50^th^, 75^th^, and 95^th^ percentiles of serum prealbumin. We further applied a two-piecewise linear regression model to examine the threshold effect of the serum prealbumin on all-cause mortality using a smoothing function (25, 26).

To ensure the stability of the results, we conducted two sensitivity analyses. In one, we repeated the analysis after excluding patients who died 3 months after follow-up began in order to avoid confounding due to premature death. In another, we separately examined patients with or without liver cirrhosis.

We performed all statistical analyses using R version 3.5.1 (https://www.r-project.org/). All P values were two-tailed, with a P value under 0.05 indicating statistical significance.

## Results

### Baseline Characteristics

The patient records contained details of 2,060 patients with HCC who underwent curative hepatectomy at our institution during January 2006 to January 2016. Based on the inclusion criteria, 2,022 (98.1%) patients were enrolled. The mean age (SD) was 49.5 (11.2) years, and 1,739 patients (86.0%) were male. Among the 2,022 patients, 31% underwent major hepatectomy, while 69% underwent minor hepatectomy. During a median follow-up (range) of 59 months (3–115 months), 385 patients (19%) were lost to follow-up. Among the remaining patients, tumor recurrence and liver failure were the main causes of mortality. The mean level of serum prealbumin level (SD) was 195.5 (69.8) mg/L, and the median (interquartile range) was 193.5 (147.0–239.0) mg/L. **Table 1** presents the characteristics of the patients stratified by quartiles of serum prealbumin.

**Table 1:** Baseline characteristics by serum prealbumin quartiles.

Characteristic	Overall (n = 2,022)	Serum prealbumin quartiles (mg/L)				P value*
		Q1 (<147, n = 501)	Q2 (147~194, n = 510)	Q3 (194~239, n = 502)	Q4 (>239, n = 509)	
Age, y						0.004
≤60	1,683 (83.2)	391 (78.0)	433 (84.9)	422 (84.1)	437 (85.9)	
>60	339 (16.8)	110 (22.0)	77 (15.1)	80 (15.9)	72 (14.1)	
Gender						<0.001
Male	1,739 (86.0)	390 (77.8)	423 (82.9)	453 (90.2)	473 (92.9)	
Female	283 (14.0)	111 (22.2)	87 (17.1)	49 (9.8)	36 (7.1)	
Liver cirrhosis						0.044
No	863 (42.7)	204 (40.7)	202 (39.6)	214 (42.6)	243 (47.7)	
Yes	1,159 (57.3)	297 (59.3)	308 (60.4)	288 (57.4)	266 (52.3)	
Tumor number						0.067
≤3	1,887 (93.3)	455.0 (90.8)	480 (94.1)	470 (93.6)	482 (94.7)	
>3	135 (6.7)	46 (9.2)	30 (5.9)	32 (6.4)	27 (5.3)	
Tumor size, cm						<0.001
≤5	825 (40.8)	129 (25.7)	182 (35.7)	228 (45.4)	286 (56.2)	
>5	1,197 (59.2)	372 (74.3)	328 (64.3)	274 (54.6)	223 (43.8)	
BCLC stage						<0.001
0/A	1,282 (63.4)	272 (54.3)	312 (61.2)	339 (67.5)	359 (70.5)	
B	328 (16.2)	92 (18.4)	76 (14.9)	79 (15.7)	81 (15.9)	
C	412 (20.4)	137 (27.3)	122 (23.9)	84 (16.7)	69 (13.6)	
Tumor capsule						0.133
Complete	1,289 (63.7)	316 (63.1)	313 (61.4)	314 (62.5)	346 (68.0)	
Incomplete	733 (36.3)	185 (36.9)	197 (38.6)	188 (37.5)	163 (32.0)	
HBsAg						<0.001
Negative	226 (11.2)	29 (5.8)	47 (9.2)	69 (13.7)	81 (15.9)	
Positive	1,796 (88.8)	472 (94.2)	463 (90.8)	433 (86.3)	428 (84.1)	
ALB, g/L						<0.001
≤35	217 (10.7)	153 (30.5)	33 (6.5)	19 (3.8)	12 (2.4)	
>35	1,805 (89.3)	348 (69.5)	477 (93.5)	483 (96.2)	497 (97.6)	
AST, U/L						<0.001
≤40	1,082 (53.5)	181 (36.1)	253 (49.6)	300 (59.8)	348 (68.4)	
>40	940 (46.5)	320 (63.9)	257 (50.4)	202 (40.2)	161 (31.6)	
ALT, U/L						0.290
9≤40	1,198 (59.2)	283 (56.5)	305 (59.8)	293 (58.4)	317 (62.3)	
>40	824 (40.8)	218 (43.5)	205 (40.2)	209 (41.6)	192 (37.7)	
TBIL, μmol/L						<0.001
≤21	1,812 (89.6)	421 (84.0)	459 (90.0)	465 (92.6)	467 (91.7)	
>21	210 (10.4)	80 (16.0)	51 (10.0)	37 (7.4)	42 (8.3)	
Child-Pugh						<0.001
A	1,974 (97.6)	470 (93.8)	505 (99.0)	493 (98.2)	506 (99.4)	
B	48 (2.4)	31 (6.2)	5 (1.0)	9 (1.8)	3 (0.6)	
AFP, ng/ml						<0.001
≤400	1,174 (58.1)	252 (50.3)	289 (56.7)	304 (60.6)	329 (64.6)	
>400	848 (41.9)	249 (49.7)	221 (43.3)	198 (39.4)	180 (35.4)	

Data are n (%). Q, quartile; AFP, alpha-fetoprotein; ALB, serum albumin; ALT, alanine transaminase; AST, aspartate aminotransferase; BCLC, Barcelona Clinic Liver Cancer; HBsAg, hepatitis B surface antigen; TBIL, total bilirubin.

*Pearson’s chi-squared test or Fisher’s exact test, P value < 0.05 indicates a significant difference between quartiles.

### Cox Regression Analysis

To assess whether clinical variables contributed to the risk of all-cause mortality, we conducted a univariate Cox regression analysis of clinical characteristics (**Table 2**). We found that tumor size and number, BCLC stage, tumor capsule, ALB, AST, ALT, and AFP levels were related to the risk of all-cause mortality. In contrast, age, sex, liver cirrhosis, HBsAg, TBIL, and Child-Pugh were not related to the risk of all-cause mortality in our cohort. To control for any possible confounding factors affecting the association between serum prealbumin and the risk of all-cause mortality, subsequent multivariate Cox regression and RCS models were adjusted for age, sex, tumor size and number, tumor capsule, AFP, HBsAg, ALB, AST, ALT, TBIL, Child-Pugh, liver cirrhosis, and BCLC stage.

**Table 2:** Risk of mortality associated with serum prealbumin*.

Variables	HR (95% CI)	P value
Age, y (>60 *vs ≤*60)	1.10 (0.92–1.32)	0.299
Sex (F *vs* M)	0.81 (0.66–1.01)	0.061
Liver cirrhosis (Yes *vs* No)	1.09 (0.95–1.26)	0.234
Tumor number (>3 *vs ≤*3)	2.20 (1.75–2.78)	<0.001
Tumor size,cm (>5 *vs ≤*5)	2.31 (1.98–2.70)	<0.001
BCLC stage		
B *vs* 0/A	1.93 (1.60–2.33)	<0.001
C *vs* 0/A	3.97 (3.39–4.66)	<0.001
Capsule (Incomplete *vs* Complete)	1.88 (1.63–2.16)	<0.001
HBsAg (Positive *vs* Negative)	1.04 (0.83–1.29)	0.755
ALB, g/L (>35 *vs ≤*35)	0.64 (0.52–0.79)	<0.001
AST, U/L (>40 *vs ≤*40)	1.99 (1.73–2.29)	<0.001
ALT, U/L (>40 *vs ≤*40)	1.32 (1.14–1.51)	<0.001
TBIL, μmol/L (>21 *vs ≤*21)	1.07 (0.85–1.34)	0.588
Child-Pugh B *vs* A	1.52 (1.01–2.28)	0.043
AFP, ng/ml (>400 *vs ≤*400)	1.48 (1.29–1.71)	<0.001

Data are HR (95% CI), unless otherwise specified.

*HRs for risk of all-cause mortality, and the corresponding 95% CIs were calculated by Cox proportional hazards regression model.

We analyzed the associations between serum prealbumin level and the risk of all-cause mortality (**Table 3**). In model 1, which was adjusted for age and sex, the HRs (95% CIs) of the risk of all-cause mortality across increasing quartiles of serum prealbumin were 1.00 for Q1, 0.68 (0.57–0.82) for Q2, 0.50 (0.41–0.60) for Q3, and 0.37 (0.30–0.45) for Q4 (P for trend < 0.001). The HR was 0.68 (0.63–0.73) per 1-SD increase in serum prealbumin. According to model 2, the HRs (95% CIs) were 1.00 for Q1, 0.78 (0.64–0.95) for Q2, 0.66 (0.53–0.81) for Q3, and 0.51 (0.41–0.64) for Q4 (P for trend < 0.001). In model 2, the HR was 0.77 (0.71–0.84) per 1-SD increase in serum prealbumin. Inclusion of a quadratic term for serum prealbumin in the Cox proportional hazard models suggested a linear correlation between serum prealbumin and all-cause mortality. However, RCS analysis showed this correlation to be non-linear.

**Table 3:** Risk of mortality associated with serum prealbumin.

	Serum prealbumin quartiles (mg/L)				Per-SD	P for trend*
	Q1 (<147)	Q2 (147~194)	Q3 (194~239)	Q4 (>239)		
All patients						
Case	501	510	502	509	2,022	–
No. of death	259 (51.7%)	209 (41.0%)	176 (35.1%)	146 (28.7%)	790 (39.1%)	–
Model 1^†^	1.00 (ref)	0.68 (0.57–0.82)	0.50 (0.41–0.60)	0.37 (0.30–0.45)	0.68 (0.63–0.73)	<0.001
Model 2^‡^	1.00 (ref)	0.78 (0.64–0.95)	0.66 (0.53–0.81)	0.51 (0.41–0.64)	0.77 (0.71–0.84)	<0.001
Patients with OS >3 months						
Case	448	476	473	486	1,883	–
No. of death	229 (51.1%)	194 (40.8%)	165 (34.9%)	138 (28.4%)	726 (38.6%)	–
Model 1^†^	1.00 (ref)	0.70 (0.58–0.85)	0.52 (0.42–0.63)	0.38 (0.31–0.47)	0.70 (0.64–0.75)	<0.001
Model 2^‡^	1.00 (ref)	0.80 (0.65–0.98)	0.64 (0.52–0.80)	0.49 (0.39–0.63)	0.77 (0.71–0.84)	<0.001

Data are HR (95% CI), unless otherwise specified. ref, reference; Q, quartile.

*Tests for linear trend were done by modeling the median value of each quartile to test ordered relations across quartiles of serum prealbumin.

^†^Adjusted for age and gender.

^‡^Adjusted for age, gender, tumor number, tumor size, tumor capsule, HBsAg, liver cirrhosis, AFP, ALB, AST, ALT, TBIL, Child-Pugh, and BCLC stage.

### Subgroup Analysis

When we analyzed serum prealbumin in correlation with all-cause mortality across subgroups of clinical characteristics (**Table 4**), we detected no significant subgroup interactions. In analyses stratified by age, sex, tumor capsule, tumor size, liver cirrhosis, HBsAg, AFP, ALT, AST, Child-Pugh, and BCLC stage, inclusion of a quadratic term for serum prealbumin in the Cox proportional hazard models revealed a linear correlation between serum prealbumin and all-cause mortality. However, this correlation was not linear in subsets of patients with tumor number >3, ALB ≤35 g/L, or TBIL >21 μmol/L (**Table 4**).

**Table 4:** Stratified associations between serum prealbumin and all-cause mortality.

Characteristic	Serum prealbumin quartiles (mg/L)				P for trend*	P for interaction
	Q1 (<147, n = 501)	Q2 (147~194, n = 510)	Q3 (194~239, n = 502)	Q4 (>239, n = 509)		
Age, y						0.516
≤60	1.00 (ref)	0.74 (0.59–0.91)	0.59 (0.46–0.74)	0.48 (0.37–0.61)	<0.001	
>60	1.00 (ref)	0.90 (0.56–1.45)	0.80 (0.49–1.30)	0.48 (0.27–0.85)	0.015	
Gender						0.452
Male	1.00 (ref)	0.78 (0.63–0.97)	0.64 (0.51–0.80)	0.50 (0.39–0.63)	<0.001	
Female	1.00 (ref)	0.68 (0.40–1.14)	0.44 (0.21–0.91)	0.21 (0.08–0.57)	<0.001	
Liver cirrhosis						0.883
No	1.00 (ref)	0.75 (0.55–1.03)	0.55 (0.39–0.77)	0.47 (0.33–0.67)	<0.001	
Yes	1.00 (ref)	0.73 (0.57–0.94)	0.62 (0.48–0.82)	0.46 (0.34–0.62)	<0.001	
Tumor number						0.098
≤3	1.00 (ref)	0.76(0.62–0.93)	0.58 (0.46–0.72)	0.46 (0.36–0.59)	<0.001	
>3	1.00 (ref)	0.62 (0.30–1.29)	0.93 (0.47–0.86)	0.51 (0.24–1.07)	0.131	
Tumor size, cm						0.373
≤5	1.00 (ref)	0.60 (0.39–0.91)	0.53 (0.34–0.80)	0.43 (0.28–0.65)	<0.001	
>5	1.00 (ref)	0.83 (0.66–1.03)	0.67 (0.52–0.85)	0.49 (0.37–0.65)	<0.001	
BCLC stage						0.881
0/A	1.00 (ref)	0.76 (0.56–1.02)	0.60 (0.44–0.82)	0.50 (0.36–0.70)	<0.001	
B	1.00 (ref)	0.70 (0.43–1.13)	0.55 (0.35–0.87)	0.44 (0.26–0.75)	0.001	
C	1.00 (ref)	0.80 (0.58–1.11)	0.65 (0.45–0.95)	0.46 (0.30–0.70)	<0.001	
Tumor capsule						0.303
Complete	1.00 (ref)	0.89 (0.68–1.16)	0.59 (0.44–0.79)	0.58 (0.43–0.79)	<0.001	
Incomplete	1.00 (ref)	0.66 (0.49–0.88)	0.63 (0.47–0.85)	0.37 (0.26–0.53)	<0.001	
HBsAg						0.614
Negative	1.00 (ref)	0.86 (0.39–1.87)	0.57 (0.27–1.17)	0.39 (0.19–0.81)	0.003	
Positive	1.00 (ref)	0.75 (0.61–0.93)	0.60 (0.48–0.75)	0.49 (0.38–0.62)	<0.001	
ALB, g/L						0.595
≤35	1.00 (ref)	0.99 (0.54–1.81)	0.44 (0.20–1.00)	0.62 (0.21–1.80)	0.082	
>35	1.00 (ref)	0.75 (0.61–0.92)	0.63 (0.50–0.78)	0.47 (0.37–0.59)	<0.001	
AST, U/L						0.559
≤40	1.00 (ref)	0.81 (0.59–1.12)	0.56 (0.40–0.79)	0.44 (0.31–0.62)	<0.001	
>40	1.00 (ref)	0.72 (0.56–0.93)	0.67 (0.51–0.88)	0.50 (0.36–0.69)	<0.001	
ALT, U/L						0.626
≤40	1.00 (ref)	0.79 (0.61–1.03)	0.56 (0.42–0.76)	0.45 (0.33–0.62)	<0.001	
>40	1.00 (ref)	0.70 (0.52–0.94)	0.67 (0.50–0.90)	0.48 (0.34–0.68)	<0.001	
TBIL, μmol/L						0.174
≤21	1.00 (ref)	0.79 (0.64–0.97)	0.60 (0.48–0.75)	0.47 (0.37–0.59)	<0.001	
>21	1.00 (ref)	0.49 (0.25–0.94)	0.72 (0.34–1.50)	0.47 (0.21–1.04)	0.091	
AFP, ng/ml						0.962
≤400	1.00 (ref)	0.77 (0.58–1.02)	0.61 (0.45–0.82)	0.48 (0.35–0.65)	<0.001	
>400	1.00 (ref)	0.78 (0.59–1.03)	0.65 (0.48–0.87)	0.48 (0.34–0.68)	<0.001	

Data are HR (95% CI), unless otherwise specified. ref, reference; Q, quartile.

*Tests for linear trend were done by modeling the median value of each quartile to test ordered relations across quartiles of serum prealbumin.

Adjusted for age, gender, tumor number, tumor size, tumor capsule, HBsAg, liver cirrhosis, AFP, ALB, AST, ALT, TBIL, Child-Pugh, and BCLC stage.

### Dose–Response Analysis

**Figure 1:**
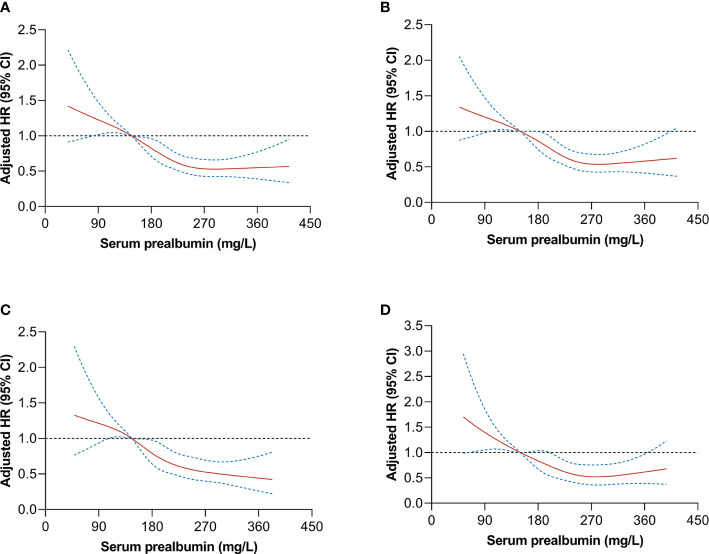
**|** Dose–response relationship between serum prealbumin and the risk of mortality after hepatectomy in patients with hepatocellular carcinoma. Graphs show the hazard ratio (HR; *solid red lines*) and 95% confidence interval (CI, *dotted blue lines*) describing the association of serum prealbumin with the risk of mortality. Cox regression analysis with a restricted cubic spline approach was conducted to allow flexible, non-linear assessment of the HR for mortality in **(A)** all patients (n = 2,022), **(B)** patients with overall survival >3 months (n = 1,883), **(C)** patients with liver cirrhosis (n = 1,159), or **(D)** patients without liver cirrhosis (n = 863) (all *P* < 0.001). All models were adjusted for age, sex, HBsAg, liver cirrhosis, tumor size and number, tumor capsule, AFP, ALB, ALT, AST, TBIL, Child-Pugh, and BCLC stage.

**Figure 2:**
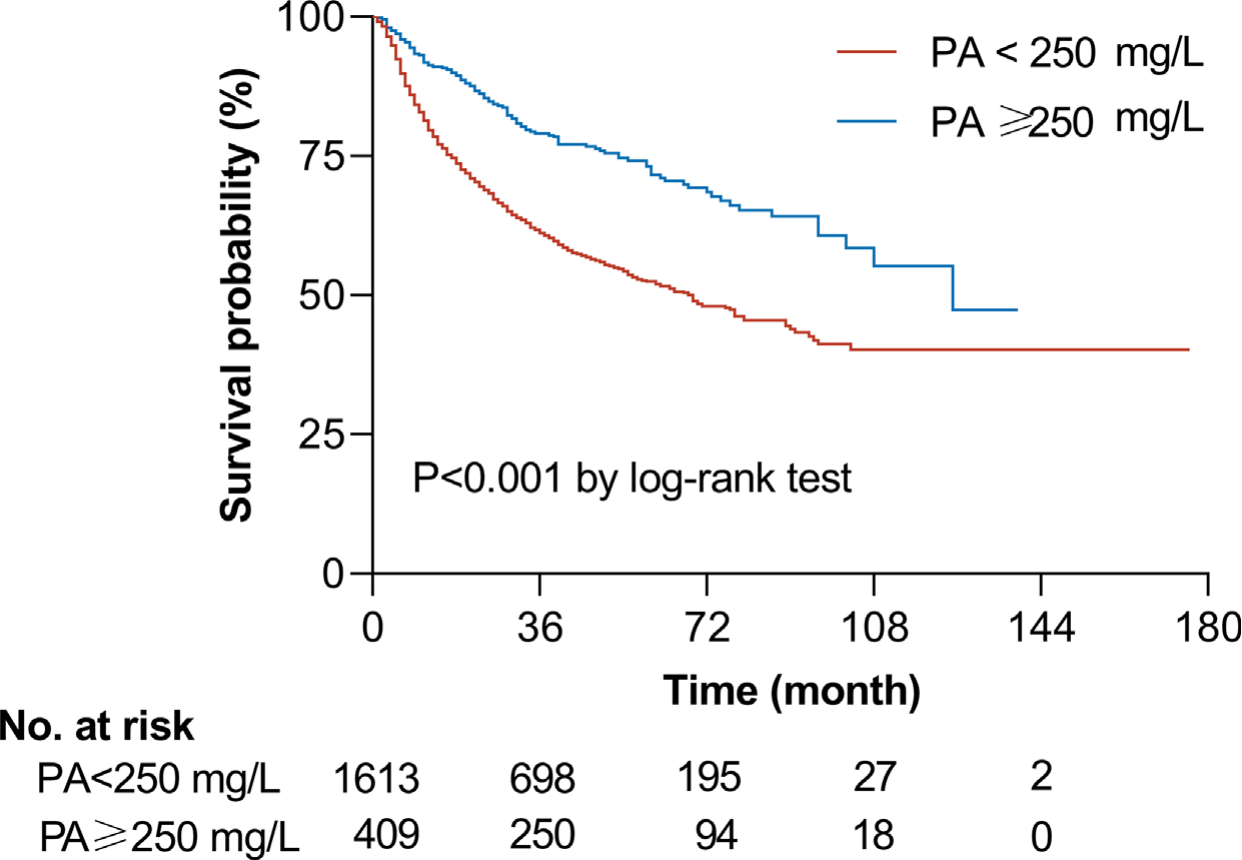
**|** Unadjusted Kaplan-Meier curve of overall survival according to serum prealbumin (PA) level. The graph shows the survival probability according to serum prealbumin level in patients with a level above a threshold of 250 mg/L (*blue line*, n = 409) or below the threshold (*red line*, n = 1,613). The two groups differed significantly in survival, based on the log-rank test. The number of patients indicated in the table is the number of patients at risk at the indicated time.

We evaluated the potential dose-response relationship between serum prealbumin and the risk of all-cause mortality using an RCS model adjusted for potential confounders. Serum prealbumin showed an L-shape, non-linear dose-response relationship with the risk of all-cause mortality (P for non-linearity <0.001, **Figure 1A**). RCS analyses based on a two-piecewise linear regression model included a knot at 250 mg/L, and Kaplan-Meier survival plots showed significantly higher all-cause mortality in patients with serum prealbumin below 250 mg/L than in those with serum prealbumin above this threshold (P < 0.001, **Figure 2**). The risk of all-cause mortality decreased with serum prealbumin level over the threshold (HR per SD decrease: 0.73, 95% CI: 0.64–0.82, **Table 5**). Risk was no longer reduced when the serum prealbumin level was above the threshold (HR 1.15, 95% CI: 0.87–1.53).

**Table 5:** Threshold effect analysis of serum prealbumin on all-cause mortality.

	Case	No. of death (%)	Per-SD	
			HR (95% CI)	P
PA<250 g/ml				
Model 1^†^	1,613	679 (42.1%)	0.64 (0.58–0.71)	<0.001
Model 2^‡^			0.73 (0.64–0.82)	<0.001
PA≥250 g/ml				
Model 1^†^	409	111 (27.1%)	1.09 (0.83–1.43)	0.533
Model 2^‡^			1.15 (0.87–1.53)	0.319

PA, Prealbumin.

^†^Adjusted for age and gender.

^‡^Adjusted for age, gender, tumor number, tumor size, tumor capsule, HBsAg, liver cirrhosis, AFP, ALB, AST, ALT, TBIL, Child-Pugh, and BCLC stage.

### Sensitivity Analysis

The exclusion of patients who died 3 months prior to follow-up did not substantially alter the results described above, suggesting that the findings are robust (**Table 3**, **Figure 1B**). Similarly, excluding patients with or without liver cirrhosis did not substantially affect the results (**Figures 1C, D**).

## Discussion

This is the first epidemiological study to investigate the dose-response association between serum prealbumin level and the risk of all-cause mortality after hepatectomy in patients with HCC. In our large cohort study, we demonstrate that serum prealbumin level is independently associated with the risk of all-cause mortality. The association between serum prealbumin level and all-cause mortality was non-linear: among patients with serum prealbumin below 250 mg/L, risk fell by 27% per SD increase of 69.8 mg/L.

Although some studies have shown that serum prealbumin is associated with the prognosis of HCC patients after hepatectomy (16, 17), no study assessed its association with the risk of all-cause mortality. After controlling for potential confounders, quadratic terms for serum prealbumin in the Cox proportional hazard models suggested a linear association between serum prealbumin and all-cause mortality. However, RCS analysis showed the association to be non-linear. These findings were not substantially affected by potential confounding from premature death or presence of liver cirrhosis. The findings of this study suggest that lower levels of serum prealbumin might be associated with increased risk of all-cause mortality in HCC patients in a non-linear manner.

Since the reference values for what is considered a “normal” serum prealbumin level vary considerably, investigating the appropriate cut-off value of prealbumin for each particular type of cancer is important for predicting OS. Serum prealbumin level using a cut-off value of 170 mg/L could predict long-term OS after hepatectomy for patients with HCC (17). We have previously shown that serum prealbumin <182 mg/L is associated with poor prognosis based on maximally selected rank statistics, and therefore we consider serum prealbumin levels ≥182 mg/L as normal. However, the lower limit of the normal reference range is 200 mg/L in many hospitals (16, 18). Based on the non-linear effects of the serum prealbumin in our study, a serum prealbumin level over 250 mg/L may be considered safe. In our cohort, all-cause mortality was significantly higher among patients with serum prealbumin below this threshold than among patients with levels above this threshold. Under this threshold, the risk of all-cause mortality decreased with increasing serum prealbumin level (HR per SD decrease: 0.73, 95% CI: 0.64–0.82).

Although how prealbumin may influence the prognosis of patients with HCC is not fully understood, a role for immunosuppression in the development of tumors has been widely accepted (27). Prealbumin promotes the production of lymphocytes, with lower serum level associated with reduced lymphocyte number, which leads to a suboptimal immune status (28). Prealbumin is an acute-phase liver protein with a half-life of only 2–3 days and can be used to reflect nutritional and inflammatory status as well as the liver’s ability to synthesize protein (29). The nutritional status is closely related to postoperative recurrence and prognosis (30). Although albumin is more commonly used than prealbumin to detect protein malnutrition and assess liver function in the clinic (11, 31, 32), some studies suggest that prealbumin is more sensitive and specific (31, 33). Therefore, serum prealbumin may be a good indicator of nutritional status and prognosis, and may be preferable to albumin for predicting the risk of death after curative hepatectomy for HCC.

The strengths of our study include the relatively large cohort, relatively long follow-up, and our adjustment for HBsAg and liver cirrhosis. Moreover, we applied an RCS model to evaluate the dose-response relationship between serum prealbumin and the risk of all-cause mortality in HCC patients. RCS models are powerful tools that can avoid the loss of information and statistical power caused by discretization of continuous variables (21). Nevertheless, our study has several limitations. First, it was retrospective, and thus, incomplete adherence to the post-resection follow-up protocol and potential confounders for OS are inevitable. In addition, although the multivariable analysis was adjusted for several covariates, we were unable to control for other unknown confounders for lack of data. For example, some inflammatory and nutritional factors such as neutrophil-lymphocyte ratio, platelet-lymphocyte ratio, and sarcopenia were not assessed (10, 34, 35). This may reduce the accuracy of our estimate of the association between serum prealbumin and all-cause mortality. On the other hand, our results are likely to be reliable given that they remained robust to several Cox regression models and sensitivity analyses. Third, we did not measure serum prealbumin in repeated samples in the same patients at different times and thus we could not correct for regression dilution, which could have led to underestimation of the association between serum prealbumin and the risk of all-cause mortality.

## Conclusions

We provide evidence that serum prealbumin is non-linearly associated with all-cause mortality after hepatectomy in HCC patients. A serum prealbumin level under 250 mg/L is a risk factor of all-cause mortality in HCC patients after hepatectomy. Our results should be confirmed and extended in pooled analyses across large prospective cohorts of HCC patients, especially in China, where this cancer is highly prevalent.

## Data Availability Statement

The original contributions presented in the study are included in the article/supplementary materials; further inquiries can be directed to the corresponding authors.

## Ethics Statement

This study was conducted in accordance with the Declaration of Helsinki guidelines, and the protocol was approved by the Ethics Commitment of our hospitals. Patients provided written informed consent before their data were analyzed.

## Author Contributions

J-HZ and LM conceived the study. R-RH, H-TL, Z-JD, X-ML, W-FG, L-NQ, XMY, and B-DX collected and analyzed the data. Z-JD and J-HZ analyzed the data. R-RH and H-TL drafted the manuscript. B-DX, L-QL, and LM revised the manuscript. All authors contributed to the article and approved the submitted version.

## Funding

This work was partly supported by the Self-Raised Scientific Research Fund of the Ministry of Health of Guangxi Province (Z2016512, Z2015621, and Z20200923), the Guangxi Natural Science Foundation (2018GXNSFBA138018, 2018GXNSFAA050124, and 2020GXNSFAA159022), the China Postdoctoral Science Foundation (2019M663876XB), the National Natural Science Foundation of China (82060510), the National Major Special Science and Technology Project (2017ZX10203207), and “Guangxi BaGui Scholars” Special Fund (2019AQ20).

## Conflict of Interest

The authors declare that the research was conducted in the absence of any commercial or financial relationships that could be construed as a potential conflict of interest.
